# Supplementary material for: Proteome Analysis of Alpine Merino Sheep Skin Reveals New Insights into the Mechanisms Involved in Regulating Wool Fiber Diameter
Source: Int J Mol Sci. 2023 Oct 16;24(20):15227. doi: 10.3390/ijms242015227 (PMC10607505; doi:10.3390/ijms242015227)
Supplement: Supplementary file 1 [file ijms-24-15227-s001.zip › Supplementary Table S1.pdf]

Table S1A. gradient elution condition

Time(min)	Gradient
0-10min	2% B
10-10.01min	2-5% B
10.01-37min	5-20% B
37-48min	20-40% B
48-48.01min	40-90% B
48.01-58min	90% B
58-58.01min	90-2% B
58.01-63min	2% B

Table S1B. DDA mass spectrometry conditions

Items	Value
Ion Mobility	0.85-1.3
Capillary	1.4KV
Mass Range	100-1700m/z
Dry Temperature	180℃
Collision Energy	20-59eV
Dry Gas	3.0L/min

Table S1C. DIA mass spectrometry conditions

Items	Value
Ion Mobility	0.7-1.3
Capillary	1.4KV
Mass Range	100-1700m/z
Dry Temperature	180℃
Collision Energy	20-59eV
Dry Gas	3.0L/min
