# Supplementary figures and images for: Proteome Analysis of Alpine Merino Sheep Skin Reveals New Insights into the Mechanisms Involved in Regulating Wool Fiber Diameter
Source: Int J Mol Sci. 2023 Oct 16;24(20):15227. doi: 10.3390/ijms242015227 (PMC10607505; doi:10.3390/ijms242015227)

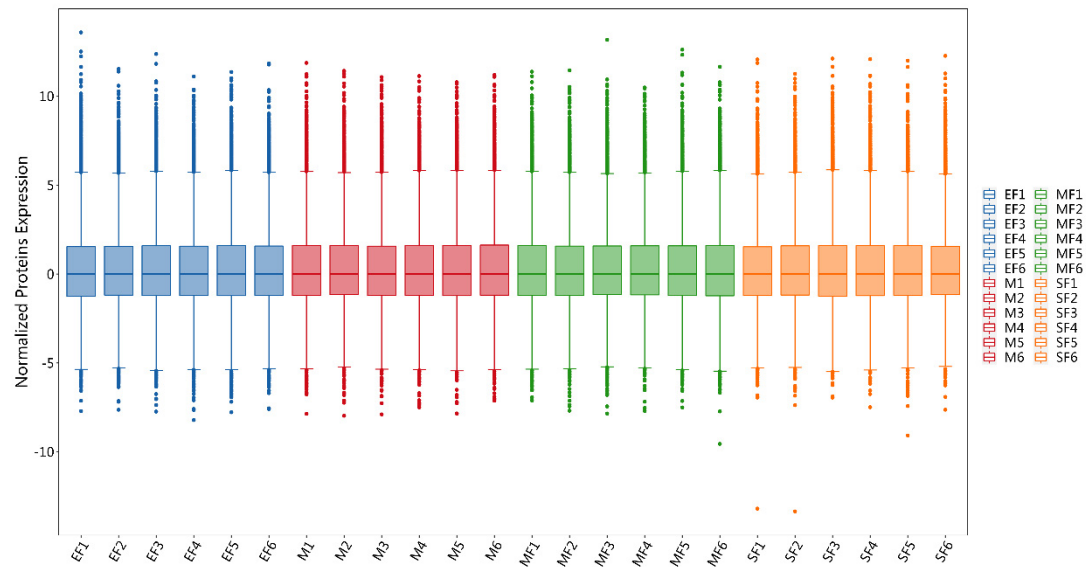

Figure S1. Expression of credible protein

Supplement: Supplementary file 1 [file ijms-24-15227-s001.zip › Supplementary Figure S1.pdf]
